# Combined Treatment of Dendrosomal-Curcumin and Daunorubicin Synergistically Inhibit Cell Proliferation, Migration and Induce Apoptosis in A549 Lung Cancer Cells

**DOI:** 10.34172/apb.2023.050

**Published:** 2022-07-02

**Authors:** Seyed Sadegh Eslami, Davod Jafari, Abbas Ghotaslou, Moein Amoupour, Amir Asri Kojabad, Rasool Jafari, Navid Mousazadeh, Parastoo Tarighi, Majid Sadeghizadeh

**Affiliations:** ^1^Student Research Committee, Faculty of Allied Medicine, Iran University of Medical Sciences, Tehran, Iran.; ^2^Department of Medical Biotechnology, Faculty of Allied Medicine, Iran University of Medical Sciences, Tehran, Iran.; ^3^Department of Clinical Laboratory Sciences, School of Allied Medical Sciences, Kashan University of Medical Sciences, Kashan, Iran.; ^4^Department of Medical Parasitology and Mycology, School of Medicine, Urmia University of Medical Sciences, Urmia, Iran.; ^5^Department of Medical Biotechnology, School of Medicine, Zanjan University of Medical Sciences, Zanjan, Iran.; ^6^Department of Molecular Genetics, Faculty of Biological Sciences, Tarbiat Modares University, Tehran, Iran.

**Keywords:** Combinatorial therapy, Cell culture, Daunorubicin, Dendrosomal curcumin, Lung cancer, Synergistic effect

## Abstract

**Purpose:**

Chemotherapy drugs used to treat lung cancer are associated with drug resistance and severe side effects. There have been rising demands for new therapeutic candidates and novel approaches, including combination therapy. Here, we aimed to investigate the combinatorial effect of a dendrosomal formulation of curcumin (DNC) and daunorubicin (DNR) on the A549 lung cancer cell line.

**Methods:**

We performed cytotoxicity, apoptosis, cell migration, colony-formation capacity, and gene expression analysis to interpret the mechanism of action for a combination of DNC and DNR on A549 cells.

**Results:**

Our results revealed that the combination of DNC and DNR could synergistically inhibit the A549 cells’ growth. This synergistic cytotoxicity was further approved by flow cytometry, migration assessment, colony-forming capacity and gene expression analysis. DNR combination with DNC resulted in increased apoptosis to necrosis ratio compared to DNR alone. In addition, the migration and colony-forming capacity were at the minimal range when DNC was combined with DNR. Combined treatment decreased the expression level of *MDR-1, hTERT* and *Bcl-2* genes significantly. In addition, the ratio of *Bax/Bcl2* gene expression significantly increased. Our analysis by free curcumin, dendrosomes and DNC also showed that dendrosomes do not have any significant cytotoxic effect on the A549 cells, suggesting that this carrier has a high potential for enhancing the curcumin’s biological effects.

**Conclusion:**

Our observations suggest that the DNC formulation of curcumin synergistically enhances the antineoplastic effect of DNR on the A549 cell line through the modulation of apoptosis/necrosis ratio, as well as *Bax/Bcl2* ratio, *MDR-1* and *hTERT* gene expression.

## Introduction

 Lung cancer is the leading cause of cancer-associated mortality in males and females worldwide.^1^ In the United States, 228 150 new cases (116 440 in men and 111 710 in women) and 142 670 deaths (76 650 in men and 66 020 in women) of lung cancer have been reported for 2019 (according to the American Cancer Society’s estimates for lung cancer).^2^ There are two major types of lung cancer: non-small cell lung cancer (NSCLC) and small cell lung cancer (SCLC). NSCLC accounts for 85% of lung cancer cases.^3^

 Chemotherapy is the main treatment approach for NSCLC and SCLC.^3^ In this regard, the chemotherapy drugs commonly used for the treatment of lung cancer patients are cisplatin, carboplatin, paclitaxel and daunorubicin. However, these chemotherapeutics are associated with treatment failure due to drug resistance and resulted in various side effects.^4,5^ Therefore, demands are increasing for new therapeutic candidates and novel approaches such as combinational chemotherapy and applying novel drug-delivery systems for improving treatment efficiency and lowering drug resistance.^6^ For this purpose, the synergistic effect in combinational chemotherapy could boost the effectiveness of a single chemotherapeutic and lessen drug resistance. In addition, the application of drug-delivery systems in combinational chemotherapy could significantly improve the pharmacokinetics of the drugs and reduce their side effects.^7^

 Daunorubicin (DNR) is an anti-tumor antibiotic that has been clinically proven for the treatment of solid and non-solid tumors.^8^ It has been indicated that DNR inhibits telomerase activity in lung cancer by increasing ceramide which induces apoptosis and inhibits cell growth. DNR interacts with DNA and inhibits DNA synthesis and DNA-dependent RNA synthesis.^9-11^ However, the clinical use of DNR is often associated with severe side effects such as cardiotoxicity. In addition, the development of the drug resistance in cancerous cells against DNR is another major challenge. To overcome these problems, the application of DNR in combination with other chemotherapy drugs reduces drug resistance and intensifies the toxicity of the combined drugs on cancer cells compared to DNR alone.^12^ DNR anticancer function is the result of its incorporation into the minor groove of DNA, resulting in an arrest in replication.^10^ In addition, DNR inhibits the topoisomerase II enzyme by stabilizing the interaction of the enzyme and DNA.^11^ DNR metabolism in the body produces and accumulates free radical species and causes DNA damage through a nonspecific approach. In this regard, DNR is cytotoxic to all proliferating cells. Although anthracyclines are used for the treatment of human leukemias, lymphomas, and multiple solid tumors, they are associated with severe side effects, including cardiotoxicity, which could lead to cardiomyopathy and heart failure.^13^ In addition to reducing drug dosage, several attempts are made to develop different formulations of DNR to minimize the side effects.^14^ Prolonged administration of DNR often leads to drug resistance in patients. Likewise, recent studies have reported the therapeutic effect of DNR in combination with other agents.^12^

 Potential pharmacological activities of phytochemicals, including antioxidant,^15^ antimicrobial,^16^ antidiabetic,^17^ anti-inflammatory,^18^ and anticancer activity are used in medicine.^19-21^ Recently, various phytochemicals such as capsaicin^22^ and curcumin^23^ have been evaluated for their anti-cancer effects. Curcumin is a yellow, polyphenol derivative of the rhizome of turmeric (*Curcuma longa*) and affects a wide variety of cellular processes through modulating different molecular targets. It has been reported that curcumin has a cytotoxic effect on cancer cell lines and has a positive effect on the inhibition of tumor growth in animal models.^24,25^ Furthermore, several studies showed that curcumin attenuates chemotherapy-induced side effects such as cardiotoxicity and neurotoxicity. Modulation of cell proliferation, enhancement of apoptosis, inhibition of nuclear factor kappa B (NF-κB), suppression of angiogenic cytokines, and reduction of B-cell lymphoma 2 (*Bcl-2*) gene expression are some of the well-studied functions of curcumin.^26^ However, low bioavailability and solubility in an aqueous medium and poor pharmacokinetic features limit the curcumin efficacy in vivo. To overcome these limitations and improve the efficacy of curcumin, nano-carriers are employed for synthesizing nano-curcumin formulations.^27-29^ Accordingly, dendrosomes were used as an efficient carrier for curcumin, called dendrosomal-curcumin (DNC). The anticancer properties of DNC in the mouse models of fibrosarcoma^30^ and colorectal cancer^26^ have been studied, and the results are remarkable. It has been reported that dendrosomes significantly improve the solubility of curcumin in an aqueous medium and facilitate its cellular uptake into target cells.^31^

 Curcumin interacts with various targets in angiogenesis, metastasis, and cell cycle pathways^24^ and plays an antineoplastic role through directing various microRNAs expression.^25^ It is worth noting that the safety of curcumin has been shown even with high doses.^32^ Due to low-safety concerns, curcumin has been widely used in combination with other drugs. Most recently, we showed potential synergistic interaction between curcumin and metformin against prostate cancer cells.^33^ Similarly, a combination of curcumin with docetaxel,^34^ metformin,^35^ 5-FU, 5-FU/oxaliplatin,^36,37^ and cisplatin^38^ in cancer cell lines showed a significant synergistic effect. In addition, strong evidence showed that curcumin enhances the treatment efficacy of anthracyclines. A combination of curcumin and doxorubicin (DOX) showed an additive effect in Hodgkin lymphoma cells^29^ by enhancing the uptake of DOX through the inhibition of ATP-binding cassettes.^39^

 The low solubility of curcumin limited its clinical application and various formulations of curcumin, such as alpha-tocopherol polyethylene glycol 1000 succinate (TPGS) formulation, liposomal curcumin^27^ and solid lipid nanoparticles have improved the kinetic profile and activity of curcumin.^28,29^ In this regard, we developed dendrosomal-curcumin (DNC) for the combination with DNR. Dendrosomes are polymeric micelle/polymersome structures introduced for the first time by Sarbolouki et al as a gene delivery system of 100 nm size.^40^

 In this study, regarding the drug resistance and cardiotoxicity of DNR,^13^ as well as positive potentials of curcumin for the side effects of drugs,^26^ we used DNC as an adjuvant for DNR. In addition, the effects of DNR, DNC, and their different combinations were evaluated on cell viability, apoptosis, gene expression, and cell migration in A549 lung cancer cells.

## Materials and Methods

###  Materials

 The human lung carcinoma cell line, A549, was obtained from the Pasteur Institute of Iran, Tehran, Iran. RPMI-1640 medium (Gibco, UK) and supplemented with 10% heat-inactivated fetal bovine serum (FBS: Gibco, Invitrogen, UK), and then used for cell culture. For the synthesis of dendrosomes, oleoyl chloride, polyethylene glycol 400 (Sigma-Aldrich, USA) and triethyl amine (Merck) were used. Methyl-Thiazol-Tetrazolium (MTT) from Sigma-Aldrich, Seelze, Germany, was used for MTT assay. MabTag’s Annexin-V Apoptosis Detection Kit (MabTag GmbH, Germany) was used for the analysis of apoptosis and cell death. Total cellular RNA was isolated with One Step-RNA extraction kit (BIO BASIC INC, Canada). DNase I s purchased from Thermo Fisher Scientific, UK. Complementary DNA (cDNA) was synthesized by Prime Script^TM^ RT reagent kit (Takara Bio Inc., Shiga, Japan). Primers were synthesized by Sinaclon, Tehran, Iran, RealQ Plus 2x Master Mix Green Without ROX^TM^ (Ampliqon, Denmark) for real-time PCR.

###  Cell culture

 The human lung carcinoma cell line, A549, was obtained from the Pasteur Institute of Iran, Tehran, Iran. For the cell culture of A549 cell line, the RPMI-1640 medium (Gibco, UK) was used. This medium was supplemented with 10% heat-inactivated fetal bovine serum (FBS: Gibco, Invitrogen, UK), 2 mM L-Glutamine, and 1% penicillin/streptomycin (100 units/mL). The A549 cultured cells were incubated at 37 °C in 5% CO_2_.

###  Dendrosomal -curcumin preparation

 Dendrosome nanoparticles and DNC were prepared based on our previous protocol.^37^ Oleoyl chloride (0.01 mol) and polyethylene glycol 400 (0.01 mol) esterification were carried out in the presence of triethyl amine (0.012 mol) at 25°C for 4 hours for the synthesis of OA400 dendrosome carrier. Chloroform was used as a solvent. After filtration of triethylamine hydrochloride salt, chloroform was eliminated from OA400 dendrosomes by evaporation in a vacuum oven at 40°C.

 To synthesize DNC, various ratios of dendrosome/Curcumin (W/W) were tested and DNC was prepared by a 25:1 ratio of dendrosome/curcumin. The absorbance of curcumin was measured through spectrophotometry.^41^ The prepared DNC solution filter was sterilized and stored at 4 °C in dark. Characterization and confirmation tests of the prepared DNC were performed according to our protocols.^30,42^

###  Cell viability assay

 Cell viability of A549 cells under drug treatment was determined by MTT assay. Briefly, 6 × 10^3^ A549 cells/well were seeded into 96 well plates with supplemented RMPI-1640 medium and were incubated overnight for the development and surface attachment of the cells. The well-attached cells were treated with different concentrations of DNR (0.1-30 μM), curcumin (5-100 μM), dendrosome (5-100 μM), DNC (5-100 μM) and the combination of DNR and DNC for 24 and 48 hours. After incubation time points, 20 μL of MTT solution (5 mg/mL) was added to each well of a 96-well plate and incubated for 4 hours at 37°C. Subsequently, the medium was removed and the formazan crystals dissolved in 100 μL of dimethyl sulfoxide (DMSO). Absorbance was measured at 570 nm with the microplate reader. The effect of each treatment was evaluated as the percentage of the viability of treated cells relative to control cells without any treatments.

###  Colony formation assay

 For the assessment of colony formation capacity of A549 cells under different treatments, a 6-well plate was used for the seeding of cells at a density of 0.4 × 10^3^ cells/well. The seeded cells were incubated overnight followed by the treatments. After 24 hours of treatment with 10 μM of DNC, 0.7 μM of DNR, and a combination of them, the medium was removed and replaced by a fresh medium and incubated for 7 days. After 7 days of incubation, the media was removed and the plate was stained with 0.1% crystal violet for 20 minutes and the colonies (with > 50 cells) were counted by light microscope.

###  Apoptosis induction assay

 MabTag’s Annexin-V Apoptosis Detection Kit was used for the analysis of the stages of apoptosis and cell death in A549 cells following the manufacturer’s instructions. Approximately, 100 × 10^3^ A549 cells/well were seeded into a 6-well plate. After 24 hours of incubation, the cells were treated with 10 μM of DNC, 0.7 μM of DNR and their combination (10 μM of DNC + 0.7 μM of DNR) for 24 hours. The treated cells were trypsinized and centrifuged at 200x g for 5 minutes. The pellet of cells was washed with medium or PBS and resuspended in 90 μL (1x) Annexin-V- binding buffer. Then, 5 μL of Annexin-V conjugate, and 5 μL of propidium iodide (PI) solutions were added to cells and incubated for 20 minutes at 15-20°C in the dark. Afterward, 400 μL of Annexin-V binding buffer (1x) was added, and the solution was centrifuged at 400 g for 5 minutes. The pellet of cells was resuspended in 200 μL (1x) Annexin-V binding buffer and immediately analyzed by flow cytometry (FACSCalibur, Becton-Dickinson). The results were analyzed with FlowJo V10 analysis software.

###  Wound healing assay

 In this assay, a 6-well plate was used for seeding A549 cells at a density of 5 × 10^4^ cells/well for the assessment of their in vitro wound healing capacity. The cells were incubated until reaching 80% confluency. A clean scrape was created through the center of the layer of cells using a sterile yellow pipette tip. In the 0, 24 and 48 h time intervals, the cells were analyzed and photos were taken under a microscope. The migration distance of cells was determined by ImageJ software.

###  RNA isolation, cDNA synthesis and real-time polymerase chain reaction (PCR)

 For gene expression analysis of A549 cells under treatments with DNC, DNR and their combination, the cells were seeded at a density of 3 × 10^5^ cells/well into a 6-well plate and treated with 10 μM of DNC, 0.7 μM of DNR and their combinations for 24 hours. Untreated cells were considered the control group. Total cellular RNA was isolated using One Step-RNA extraction kit followed by digestion with DNase I. cDNA was synthesized by PrimeScript^TM^ RT reagent kit. The synthesized cDNA was used as a template for real-time q-PCR test via Ampliqon, RealQ Plus 2x Master Mix Green Without ROX^TM^ in a light cycler (Roche Diagnostic). Quantitative RT-PCR was performed by the following steps. An initial activation step was considered for 5 minutes at 95°C. After initial activation, 45 cycles of amplification continued with a denaturation step (30 seconds at 95°C), an annealing step (15 seconds at 60°C), and an extension step (30 seconds at 72°C). The specificity of the PCR products was confirmed via a melting curve analysis. All the experiments were carried out at least in triplicate. GAPDH mRNA expression was used as a normalizer of the changes in mRNA expression level of the genes. Eventually, the 2^-∆∆CT^ method was employed for the quantification of mRNA expression fold changes. The sequences of the primers and the relevant amplicon size and genes are shown in Table 1.

**Table 1:** The sequence of the specific primers was used for real-time PCR

**Gene**	**Forward primer (5’-3’)**	**Reverse primer (5’-3’)**
*GAPDH*	CACCAGGGCTGCTTTTAACTCTGGA	CCTTGACGGTGCCATGGAATTTGC
*hTERT*	TCCATCAGAGCCAGTCTCACC	GCTGTTCACCTGCAAATCCAGA
*Bcl-2*	ATCGCCCTGTGGATGACTGAG	CAGCCAGGAGAAATCAAACAGAGG
*Bax*	GGACGAACTGGACAGTAACATGG	GCAAAGTAGAAAAGGGCGACAAC
*MDR-1*	GTCATCTTGTCCAAACTGCCTG	GTTTTGGGTTTGAGAGCCACC

###  Statistical analysis

 Statistical analysis was performed in Prism^®^ 8 software (GraphPad Software, Inc., La Jolla, CA, USA) and analyzed using one and two-way ANOVA analysis of variance followed by Tukey’s and Sidak multiple comparison tests. The statistical significance was set at *P* < 0.05. All the experiments were carried out at least in triplicate.

## Results and Discussion

###  Dendrosomal curcumin synthesis and characterization

**Figure 1:**
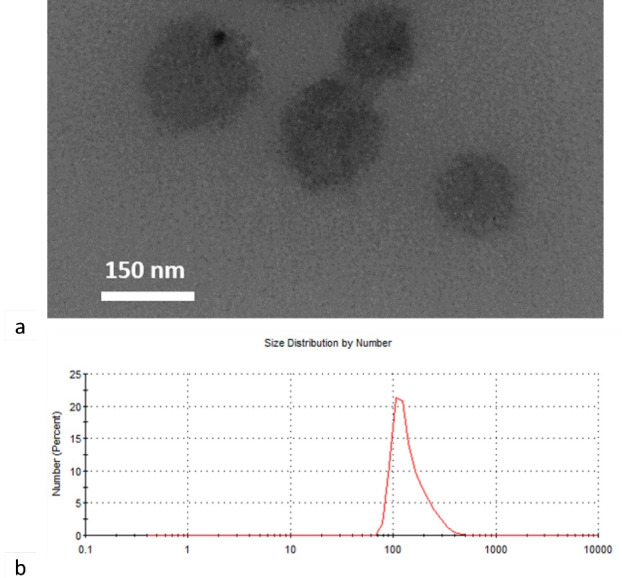
Structural characterization of DNC. a) TEM image of synthesized DNC and b) Size distribution curve of DNC obtained from DLS data

 The synthesized dendrosomes were mixed with curcumin in a certain ratio and the DNC was synthesized (Figure 1). Studies on the dendrosomal structural properties of curcumin showed a uniform size distribution of about 155 nm. DNC characterizations, such as degradation and cell uptake, have been presented in our previous studies and are not displayed here. The first report for our DNC formulation was published in 2012 by Babaei et alin which 1:25 (W/W) curcumin was encapsulated into dendrosomes. The data showed that dendrosomes significantly increased the water solubility of curcumin.^30^

###  The effect of curcumin, DNC and DNR on A549 cells viability

**Figure 2:**
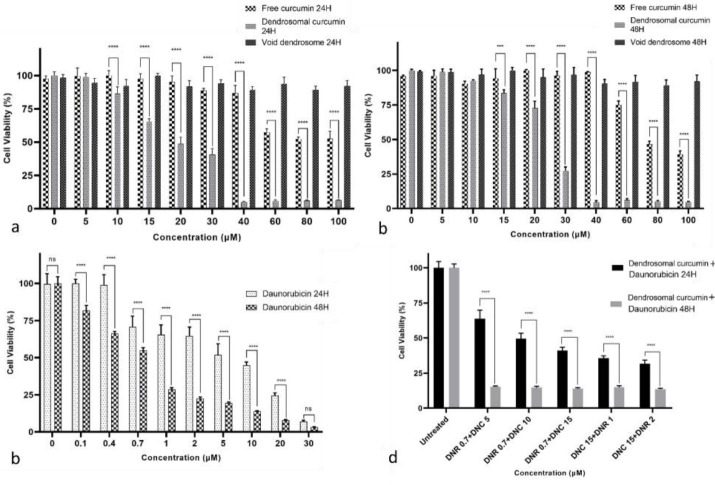
Cell viability assay (MTT) results. a and b) The cytotoxicity of free curcumin, DNC, and dendrosomes treatments, after 24 and 48 hours respectively, c) DNR treatment after 24 and 48 hours and d) combined DNC and DNR treatment after 24 and 48 hours on the A549 cells, *P* < 0.0*5* (**** *P* < 0.0001, ns: not significant)

 The cytotoxic effect of DNR, DNC, curcumin, dendrosome, and combination of DNC and DNR on the A549 cells was determined by the MTT assay (Figure 2). A549 cells were seeded into 96-well plates and treated with 0.1-30 μM DNR and 5-100 μM DNC, 5-100 μM curcumin and 5-100 μM dendrosome and analyzed after 24 and 48 hours. Based on the MTT assay results, DNC inhibited the proliferation of A549 cells compared to the control group (Figure 2a and 2b) in a dose-dependent and time-independent manner. In contrast, curcumin inhibited the proliferation of A549 cells in a dose- and time-dependent manner (Figure 2a and Figure 2b). Likewise, DNR significantly (p < 0.0001) inhibited the proliferation of A549 cells (Figure 2c). Furthermore, the half-maximal inhibitory concentration (IC50) value for DNR after 24 and 48 hours was determined by 4.043 and 0.637 μM, respectively (by GraphPad Prism 8 software). Similarly, the IC50 values calculated for DNC were 20.44 and 24.07 μM, and for curcumin the values were 91.42 and 83.17μM in 24 and 48 hours, respectively. Furthermore, no significant toxic effect was observed in dendrosome treatments. According to the mentioned results, the inhibitory effect of DNC is stronger than curcumin in the same concentrations.

 Our previous studies showed that DNC suppresses cancer cells in low doses compared to free curcumin. Besides, no significant cytotoxicity related to dendrosome carriers was observed in our previous studies.^30,37,43-52^ Similarly, in the present study, we used 5-100 µM dendrosomes and did not observe significant cytotoxicity in A549 cells. In addition, the assessment of acute and chronic cytotoxicity of DNC in BALB/c mice showed that DNC is a safe formulation even in higher concentrations.^42^

###  Evaluation of combination index 

 To indicate the impact of combined treatments, combination index (CI) values were calculated for the combined treatment of DNR and DNC. CI values demonstrate the interaction degree between DNC with DNR. For this purpose, after evaluating the effects of the compounds separately, the three different concentrations of DNR (0.7, 1, 2 μM), as well as DNC (5,10,15 μM) were selected for the combination study. After the selection of concentrations, A549 cells were treated for 24 and 48 hours. According to the MTT assay result of the combined treatments shown in Figure 2d, a combination of DNR and DNC showed a significant (*P* < 0.0001) inhibitory effect against A549 cells compared to DNR and DNC alone. In 24 hours, the combined treatment showed a dose-dependent pattern, but in 48 hours, it did not follow this pattern. However, all treatments showed a time-dependent inhibitory effect.

 The synergistic, additive, or antagonistic effect of DNR and DNC combined treatments were evaluated by CompuSyn software, version 1, based on the CI calculated by the Chou-Talalay equation (CI > 1.2, CI = 0.9–1.2 and CI < 0.9 related to an antagonistic, additive and synergistic effect, respectively).

**Figure 3:**
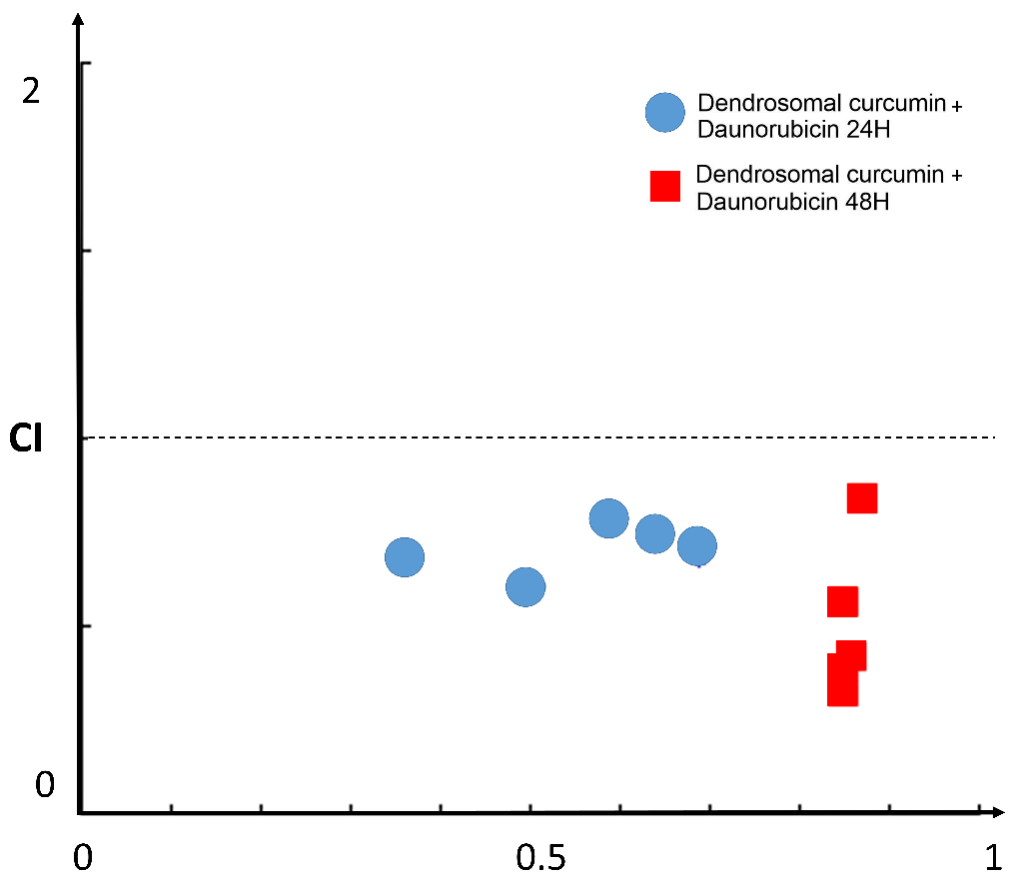
Fa-CI plot for the combination effect of DNC and DNR in 24 and 48h intervals. The points under the diagonal line suggests the synergistic effect of combined treatment, CI: Combination Index, Fa: Fraction Affected

 In this regard, the combined treatments of 0.7-2 μM DNR with 5-15 μM DNC at 24 hours were evaluated for the calculation of CI. The CI = 0.607 as the lowest CI belonged to the treatment of 0.7 μM DNR + 10 μM DNC and is associated with a complete synergistic effect. However, other doses showed a synergistic effect. In addition, for the same concentrations at 48-hour time interval, CIs for all treatments were reduced compared to CIs of 24 hours, except for the treatment of 2 μM DNR + 15 μM DNC (Table 2). Totally, all the combined treatments of DNR and DNC showed a synergistic effect in both 24- and 48-hour intervals (Figure 3).

**Table 2:** The CI data for different treatments on the A549 cells obtained with CompuSyn software

**Time point**	**DNC (μM)**	**DNR (μM)**	**Effect (Inhibition %)**	**CI**	**Interaction type**
24 h	5.0	0.7	0.36	0.68389	Synergistic
	10.0	0.7	0.5	0.60778	Synergistic
	15.0	0.7	0.59	0.79371	Synergistic
	15.0	1.0	0.64	0.74708	Synergistic
	15.0	2.0	0.69	0.70199	Synergistic
48 h	5.0	0.7	0.85	0.32941	Synergistic
	10.0	0.7	0.85	0.39010	Synergistic
	15.0	0.7	0.86	0.42722	Synergistic
	15.0	1.0	0.85	0.56595	Synergistic
	15.0	2.0	0.87	0.84179	Synergistic

 Generally, the administration of an anticancer chemotherapeutic is dose-dependent and often fails to achieve complete cancer remission owing to the heterogeneity of cancer cells and the development of multidrug resistance colonies.^53^ Further evidence shows that the combination of chemotherapeutic drugs could strongly enhance the treatment efficacy without multiplication of the toxicity.^54^ The combined treatment results in synergistic, additive or antagonistic effects. The synergistic effect is the most suitable outcome of combinational drug therapy. Recent studies used multiple methods for the interpretation of combined effects of drugs, including CI,^55^ isobolographic analysis,^56^ Bliss independence model,^57^ Loewe additivity model.^58^ However, the dose optimization and adjustments of each drug is a major effort, because different concentrations of drugs could result in different clinical outcome.^59^

###  Effects of DNC and DNR treatments on apoptosis induction in A549 cells

**Figure 4:**
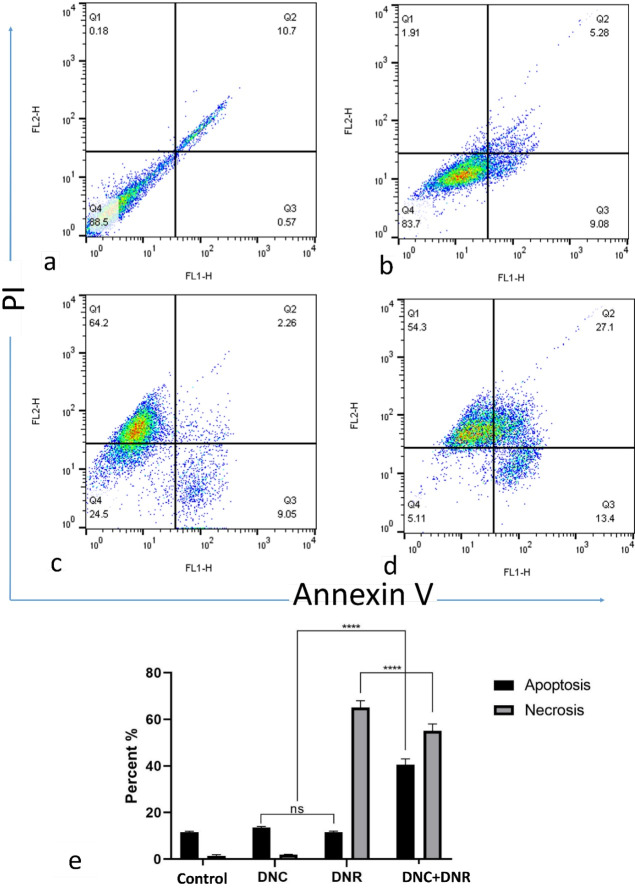
Flow cytometry diagrams of cells treated with DNC, DNR and their combination at 24 hours. a) control group without any treatment, b) DNC, c) DNR and d) combined DNC and DNR treated A549 cells. e) the proportion of apoptosis and necrosis in control, DNC, DNR and combined DNC and DNR treated A549 cells, P < 0.05 (****P < 0.0001, ns: not significant)

 A549 cells were treated with 10 μM DNC, 0.7 μM DNR, and 10, 0.7 μM of DNC + DNR, respectively (concentrations obtained based on cytotoxicity and CI analyzes) (Figure 4a-d).

 Based on the results of the apoptosis assay (in 24 hours), the lower dose of DNC did not show significant cell death. In contrast, DNR treatments showed a significant necrosis induction of about 60% compared to the control group. A combination of DNC and DNR (10 μM DNC and 0.7 μM DNR) revealed a synergistic effect on induction of apoptosis by up to 30% compared to DNC and DNR alone (*P* < 0.05) (Figure 4e). Regarding the high necrosis in DNR-treated cells, it seems that combined DNC and DNR treatment diminished the necrosis induced by DNR and simultaneously induced apoptosis. However, other biochemical assays such as caspase activation, Bid cleavage, cytochrome c release, analysis of supernatant for caspases, HMGB1, the release of cytokeratin 18 and phosphatidylserine exposure are necessary to confirm that DNR does indeed cause necrosis, and a combination of DNC and DNR shifts the cell life condition from necrosis to apoptosis. Therefore, with our analysis, it may be primarily concluded that DNC and DNR simultaneous treatment increased the induction of apoptosis, decreased necrosis, and showed a synergistic effect on the cytotoxicity to A549 cells.

###  Effects of DNC and DNR treatment on the colony formation capacity of A549 cells

**Figure 5:**
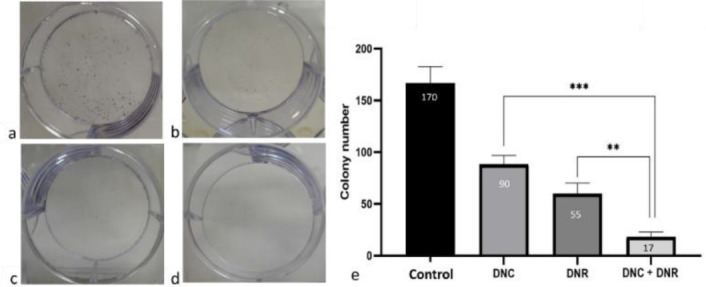
Colony forming assay results. a) control group, b) DNC, c) DNR and d) combined DNC and DNR treated A549 cells after 24 hours. e) shows the results of colony forming assay under different treatments, P < 0.05 (**P < 0.01, *** P < 0.001). Cnt: Control, DNC: Dendrosomal Curcumin and DNR: Daunorubicin

 Colony formation assay results demonstrated that the proliferation rate and colony numbers of the A549 cells treated with DNC, DNR and their combination were significantly decreased compared to the control group (*P* < 0.05) (Figure 4a-d). In addition, the inhibitory effect of the combined treatment of DNC and DNR was significantly (*P* < 0.05) higher compared to their separate treatments (Figure 5e).

 Our colony-formation assay showed that all the treatment groups (DNC, DNR, DNC + DNR) reduce clonogenic capacity of A549 cells but the minimum colony-forming capacity was observed in DNC + DNR treated cells through a synergistic effect. Furthermore, we investigated whether cell migration could be affected by DNC, DNR, and DNC + DNR.

###  Effects of DNC and DNR treatment on Bax, Bcl-2, hTERT, MDR-1 genes mRNA expression level 

**Figure 6:**
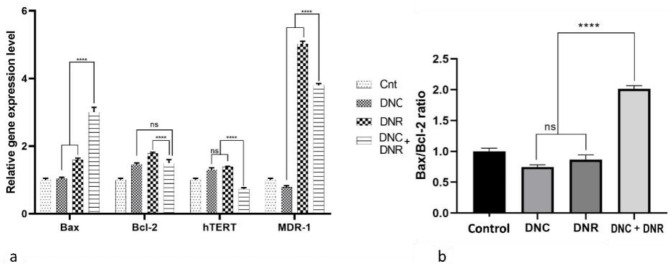
Gene expression analysis results. a) expression level of *Bax*, *Bcl-2*, *hTERT* and *MDR-1* genes in A549 cells treated with DNC, DNR and their combination as well as control group without any treatment. b) the ratio of Bax/Bcl-2 expression level as an indicator of apoptosis in DNC, DNR and their combination as well as control group without any treatment, *P* < 0.0*5* (****P < 0.0001, ns: not significant)

 The Real-time qPCR test was run to indicate the effects of the treatment of 10 μM DNC, 0.7 μM DNR, and 10, 0.7 μM DNC­­­­ + DNR, respectively at 24 hours intervals on the mRNA expression levels of *Bax*, *Bcl-2*, *hTERT* and *MDR-1* genes in A549 cells. Analysis of the results showed an 0.60 and a ~2-fold increase in the expression level of the *Bax* gene in the cells treated with DNR and a combination of DNC and DNR. In contrast, DNC treatment did not show a statistically significant change in *Bax* gene expression compared to the control group. Furthermore, *Bcl-2* gene expression was increased (*P* < 0.05) in all treatment groups (Figure 6a).

 For evaluating apoptosis at the molecular level, the *Bax*/*Bcl-2* expression ratio was assessed as a suitable indicator of apoptosis. We observed that the ratio increased only in the combined treatment of DNC and DNR (*P* < 0.0001) in comparison with single drugs. Also, the combination treatment ratio rose by one-fold compared to the control group, which is consistent with the results of our flow cytometry analysis and previous studies.

 In this study, changes in the expression of the *MDR-1* gene were studied. In DNC treated cells, the expression of this gene decreased, which was not statistically significant. In contrast, in DNR-treated cells, the expression of the *MDR-1* gene increased 4-fold compared to the control group. Eventually, in the cells treated with DNC + DNR, the expression of this gene was reduced by about 1.2-fold compared to the DNR group. The effect of DNC to *MDR-1* gene expression at the transcriptional level could lower the level of MDR pumps on the plasma membrane of cancer cells and mitigate drug resistance to DNR.^60-62^ In addition, our findings regarding the *MDR-1* gene expression could be confirmed by a previous study that provided evidence for reversing the multidrug resistance by co-delivery of DOX and curcumin.^63^

 The telomerase coding gene, *hTERT*, is activated in most human tumors^64^ and results in uncontrolled growth and proliferation of cancer cells.^65^ Targeting the activity of this enzyme can reduce cancer cell progression.^66^ Previously, we showed that curcumin downregulates the *hTERT* gene through TGF-B pathway.^45^ In the present study, the expression analysis of *hTERT *gene significantly decreased (*P* < 0.05) in the DNC + DNR treated cells. Interestingly, low doses of DNC and DNR enhanced the activity of this enzyme. Interestingly, while DNC and DNR individually increased the expression of *hTERT *gene, a combination of two drugs decreased its expression, which could consequently inhibit the progressiveness feature of cancer cells. Our results represent that the combined treatment effect on *hTERT* expression reduction is higher compare to control, DNC, and DNR alone (Figure 6b).

 In general, the results of qRT-PCR support the results of the combined treatment of DNC and DNR with MTT, apoptosis, wound healing, and colony formation assays for efficient inhibition of A549 cells by reducing the expression of *Bcl-2*, *hTERT,* and *MDR-1* as well as increasing the ratio of the *bax /bcl-2 *and *Bax*gene expression.^67^

###  Effect of DNC and DNR treatment on migration capacity in A549 cells

 We investigated whether A549 cells migration could be affected by DNC and DNR alone and combined treatments. For this purpose, A549 cells were treated with DNC and DNR and their combination (DNC and DNR) at the same concentrations of previous assays. The scratches widths were measured at different time points by ImageJ software.

**Figure 7:**
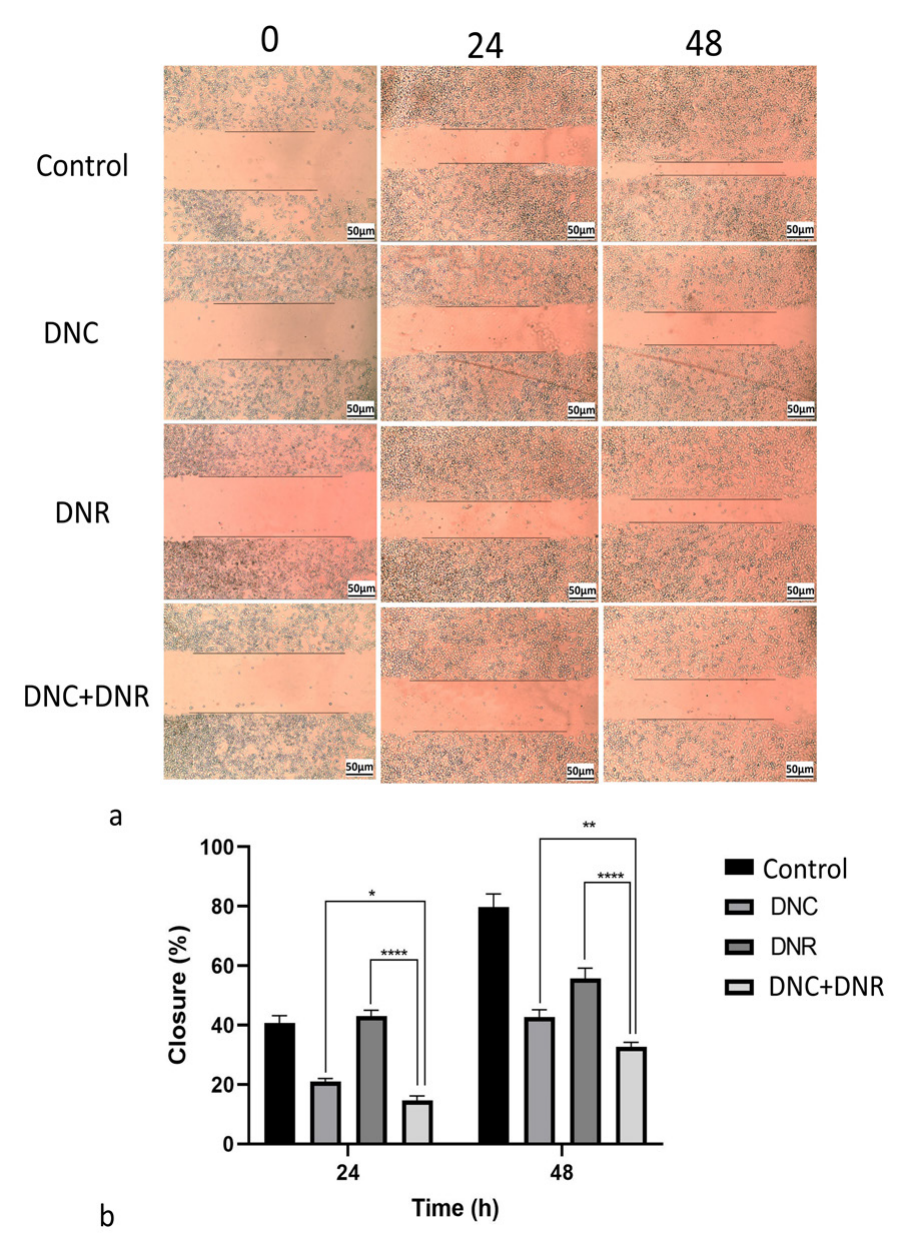
In vitro wound healing assay and cell migration results. a) the in vitro wound healing and migration capacity of control group without any treatments, DNC, DNR and combined DNC and DNR treated A549 cells in three time point (0, 24 and 48), and b) the pooled results of migration in response of DNC, DNR and their mix in A549 cells, P < 0.05 (* P < 0.05, ** P < 0.01, **** P < 0.0001 )

 Our wound healing assay demonstrated that DNC treatment decreases the migration of cells more than the control group and DNR-treated cells (Figure 7a). According to Figure 6, the combined treatment of DNC and DNR led to a significantly higher inhibition in cell migration compared to the other treatments. The results of the analysis of the effect of treatments on migration capacity have been shown in Figure 7b. Based on the results depicted in Figure 6a, the DNC and DNR combined treatment is the most effective treatment for the inhibition of A549 cells migration. As a malignant feature of high metastatic cancer, cell migration reduction may decrease the metastatic feature of NSCLC. According to the other studies on the A549 cell line and focusing on their results of wound healing, cell motility of A549 can be reversed by *mAChR3*, *EGFR*, *c- Srcand* genes inhibitor, or MMP-7 neutralizing antibody.^68^ Other studies also suggested that *mAChR3* activation induces cell migration and invasion in multiple cancers.^69-71^ It was also shown that the overexpression of these genes in NSCLC cells elevates the cancer progress.^72^ However, different and complex pathways are involved in the migration of cancer cells and tumor metastasis, and further studies are needed for the exact mechanism and pathways underlying the decreased migration potential of DNC + DNR treated A549 cell line.

 Eventually, a limitation of our study is the analysis of these combinations and formulation of the exact ratio of DNC to DNR in other lung cancer cell lines including HLC-1. In addition, in vivo experiments on animal model of lung cancer could further approve the results found in this study.

## Conclusion

 In this study, we showed that the combination of the DNC and DNR could dramatically inhibit the growth, induce apoptosis and reduce the cell migration of A549 cells in a dose- and time-dependent manner compared to single treatment of DNC and DNR. In addition, the drug resistance due to DNR treatment was lowered in the combined treatment of DNC and DNR. It could be concluded that the synergistic effect of the cytotoxicity of DNC + DNR treatment on A549 cells is associated with the downregulation of *hTERT* and *MDR-1* genes as well as an increase in the ratio of *Bax*/*Bcl-2* gene expression. Due to obvious limitations of curcumin, we used an improved formulation of curcumin (DNC). DNC shows better solubility and uptake and consequently higher toxicity than free curcumin at similar doses. We used a minimal dose of DNC as an adjuvant for DNR and the interaction of the two compounds was observed as a strong synergism. The use of DNC as an adjuvant for DNR increased the toxicity, and apoptosis instead of necrosis in A549 cells.

## Competing Interests

 Authors declare that they have no conflict of interest.

## Ethical Approval

 This research approved by ethics committee of Iran University of Medical Sciences (Ethics No. IR.IUMS.REC.1398.989).

## Funding

 This study was funded by Student Research Committee, Iran University of Medical Sciences (Grant Number: 14878).
